# Accuracy of Hemolyzed Potassium Levels in the Emergency Department

**DOI:** 10.5811/westjem.2020.8.46812

**Published:** 2020-10-20

**Authors:** Matt Wilson, Sam Adelman, JB Maitre, Joe Izzo, Ronald Valencia, Mike Yang, Jeff Dubin, Munish Goyal

**Affiliations:** Medstar Washington Hospital Center, Department of Emergency Medicine, Washington DC

## Abstract

**Introduction:**

In the emergency department (ED), pseudohyperkalemia from hemolysis may indirectly harm patients by exposing them to increased length of stay, cost, and repeat blood draws. The need to repeat hemolyzed potassium specimens in low-risk patients has not been well studied. Our objective was to determine the rate of true hyperkalemia among low-risk, adult ED patients with hemolyzed potassium specimens.

**Methods:**

We conducted this prospective observational study at two large (129,000 annual visits) academic EDs in the mid-Atlantic. Data were collected from June 2017–November 2017 as baseline data for planned departmental quality improvement and again from June 2018–November 2018. Inclusion criteria were an initial basic metabolic panel in the ED with a hemolyzed potassium level > 5.1 milliequivalents per liter that was repeated within 12 hours, age (≥18, and bicarbonate (HCO_3_) > 20. Exclusion criteria were age > 65, glomerular filtration rate (GFR) < 60, creatine phosphokinase > 500, hematologic malignancy, taking potassium-sparing or angiotensin-acting agents, or treatment with potassium-lowering agents (albuterol, insulin, HCO_3_, sodium polystyrene sulfonate, or potassium-excreting diuretic) prior to the repeat lab draw.

**Results:**

Of 399 encounters with a hemolyzed, elevated potassium level in patients with GFR ≥ 60 and age > 18 that were repeated, we excluded 333 patients for age > 64, lab repeat > 12 hours, invalid identifiers, potassium-elevating or lowering medicines or hematologic malignancies.This left 66 encounters for review. There were no instances of hyperkalemia on the repeated, non-hemolyzed potassium levels, correlating to a true positive rate of 0% (95% confidence interval 0–6%). Median patient age was 46 (interquartile range [IQR] 34 – 56) years. Median hemolyzed potassium level was 5.8 (IQR 5.6 – 6.15) millimoles per liter (mmol/L), and median repeated potassium level was 3.9 (IQR 3.6 – 4.3) mmol/L. Median time between lab draws was 145 (IQR 87 – 262) minutes.

**Conclusion:**

Of 66 patients who met our criteria, all had repeat non-hemolyzed potassiums within normal limits. The median of 145 minutes between lab draws suggests an opportunity to decrease the length of stay for these patients. Our results suggest that in adult patients < 65 with normal renal function, no hematologic malignancy, and not on a potassium-elevating medication, there is little to no risk of true hyperkalemia. Further studies should be done with a larger patient population and multicenter trials.

## INTRODUCTION

Hyperkalemia is a major concern in the clinical setting due to its life-threatening effects on skeletal muscle and risk of cardiac arrhythmia secondary to impaired neuromuscular transmission. Accordingly, evaluation and treatment of hyperkalemia is treated as a priority in the emergency department (ED). However, many blood sample specimens report a falsely elevated potassium level from hemolysis during the collection process. In 1958 Hartmann et al first reported this finding as pseudohyperkalemia, an elevation of measured potassium levels in the absence of clinical evidence of electrolyte imbalance.^1^ Pseudohyperkalemia most commonly occurs due to variability in venipuncture, including the use of tourniquets, repeated fist clenching, and sheer trauma that results in hemolysis.^2^,^3^ Hemolysis is reported to occur frequently, with one ED-based study reporting 32% of all samples had some degree of hemolysis.^3^

In the presence of a high potassium level due to hemolysis, clinicians often repeat the test to confirm a normal potassium level, which can lead to increased length of stay, multiple blood draws, increased use of healthcare resources, and needless extra risk for patients. Pseudohyperkalemia is of particular concern in the busy setting of the ED as it requires timely management and resource use until proven to not be a true emergency. The need to repeat hemolyzed potassium specimens in low-risk patients has not been well studied; there is only one prior observational study in the published literature. Khodorkovsky et al found that among a convenience sample of 42 patients with hyperkalemia from a hemolyzed specimen, glomelular filtration rate (GFR) ≥60 and normal electrocardiogram (ECG) had a 100% negative predictive value of true hyperkalemia.^4^ Our objective was to determine the rate of true hyperkalemia among low-risk adult ED patients with hemolyzed potassium specimens from a larger sample size at our institution. We hypothesized that for patients with hemolyzed potassium samples but normal renal function a priori selected criteria could exclude true hyperkalemia in 100% of cases.

## METHODS

We conducted this prospective observational study at two large (129,000 combined annual visits) academic EDs in the mid-Atlantic region. Background hemolysis rate was known to be 0.28% of chemistry samples at the institution. Data collection was approved by the institutional review board as part of a quality improvement initiative observing departmental management practices for hyperkalemia. Data were collected by an automated electronic health record (EHR) search algorithm to identify all patients meeting inclusion criteria from the period June 2017–November 2017 and again from June 2018–November 2018. Exclusion criteria were recorded for all charts and reviewed by consensus among all the physician authors to determine exemption. These criteria were intentionally limiting in order to produce a highly sensitive decision rule (Table 1).

Inclusion criteria were an initial basic metabolic panel in the ED with a hemolyzed potassium level > 5.1 milliequivalents per liter that was repeated within 12 hours, age > 18, and bicarbonate (HCO_3_) > 20. Exclusion criteria were defined a priori as age > 65, lab calculated estimated GFR < 60, creatine phosphokinase > 500, hematologic malignancy, taking potassium-sparing or angiotensin-acting agents, or treatment with potassium-lowering agents (albuterol, insulin, HCO_3_, sodium polystyrene sulfonate, or potassium-excreting diuretic) prior to the repeat lab draw. These criteria were felt by consensus at our institution to carry a historically elevated risk of hyperkalemia that would require a repeat measurement in the setting of potential hyperkalemia. We used an Excel spreadsheet (Microsoft Corporation, Redmond, WA) for descriptive statistics to evaluate our primary outcome: the rate of true hyperkalemia in adults without clinical risk factors for hyperkalemia. Pearson correlation coefficient was used to assess trend associations.

**Population Health Research Capsule**

What do we already know about this issue?

Hemolyzed potassium levels often require repeat blood draw in the ED, even in lower risk patients such as those <65 with normal renal function and no malignancy

What was the research question?

What is the rate of true hyperkalemia among lower risk adult ED patients with hemolyzed potassium specimens?

What was the major finding of the study?

Of 66 patients who met our pre-defined low-risk criteria, all had repeat potassiums within normal limits.

How does this improve population health?

*It may be possible to safely avoid a repeat blood draw in appropriately selected ED patients, thereby decreasing pain, cost, and length of stay*.

## RESULTS

**Figure 1:**
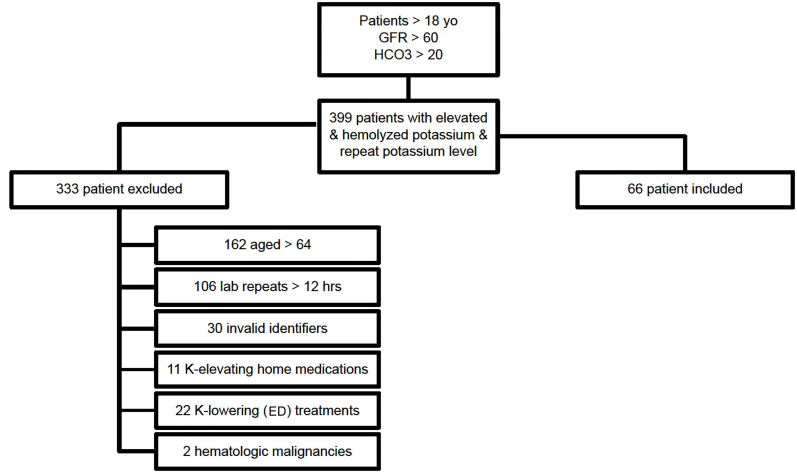
Inclusion flow chart demonstrating 399 patients with elevated potassium levels seen in hemolyzed lab draw. *GFR*, glomerular filtration rate; *K*, potassium; *ED*, emergency department.

As presented in Figure 1, there were 399 encounters with a hemolyzed, elevated potassium level in patients with GFR > 60 and age > 18 that had a repeat potassium draw during the study period. We excluded 162 patients for age > 64, 106 patients for lab repeat > 12 hours, 30 patients for duplicate and thus invalid account identifiers, 11 patients on potassium-elevating medicines, 22 patients treated with potassium-lowering medicines, and 2 patients with hematologic malignancies. This left 66 encounters after applying exclusion criteria. There were no instances of hyperkalemia on the repeated, non-hemolyzed potassium levels, correlating to a true rate of hyperkalemia of 0% (95% confidence interval 0–6%). There was no correlation between the magnitude of elevation of the hemolyzed sample and subsequent true potassium level upon repeat (r^2^ = 0.005)

Study demographics were as follows: Median patient age was 46 (interquartile range [IQR] 34 – 56) years. Median hemolyzed potassium level was 5.8 (IQR 5.6 – 6.15) millimoles per liter (mmol/L), and median repeated potassium level was 3.9 (IQR 3.6 – 4.3) mmol/L. Median time between lab draws was 145 (IQR 87 – 262) minutes.

## DISCUSSION

Of 66 patients who met our pre-specified exclusion criteria, all had repeat non-hemolyzed potassiums within normal limits. This supports our hypothesis that in appropriately selected adult ED patients < 65 years of age with normal renal function, no hematologic malignancy, and not on potassium-modulating drugs, there is no risk of true hyperkalemia. This contrasts to higher risk populations such as the large inpatient/outpatient cohort described in 2009 by Einhorn et al who associated chronic kidney disease, and ACE-inhibitor use with a hyperkalemic event rate of 3.2% and with excess mortality.^5^ This underlines the importance of safely identifying ED patients who do not need further evaluation of their pseudohyperkalemia. Khodorkovsky’s prior report from an ED setting similiarly identified a safe cohort for rapid disposition without repeating potassium levels; their criteria required use of ECG testing for evaluation.^4^ In contrast, our dataset was derived using easily accessible historical criteria without the need to assess variation in observer interpretation of ECG findings while still maintaining a negative predictive value of 100%.

An additional strength worth noting for our study is its ready applicability to the ED practice setting and ability to implement our findings for patient workflow improvement. The median of 145 minutes between lab draws suggests a ready opportunity to decrease the length of stay by speeding ED disposition for patients. It also suggests a safe way to decrease associated pain from intravenous sticks as well as healthcare-associated costs and potential harms.

## LIMITATIONS

Limitations to the study include its non-interventional and observational nature as opposed to a randomized study. This was because we felt it to be standard of care to repeat potassium levels at the time but note that in chart review 35 cases were found to have been dispositioned without repeating a pseudo-elevated potassium draw and were thus excluded. These cases presumably reflect some level of physician comfort in dispositioning these low-risk patients without a repeat lab draw. This study contributes to evidence that in appropriately selected patients this can be a safe practice. Additionally, while the study was limited to two similar, tertiary care academic EDs, it was not limited to only one center and did include a large sample size for review with broad inclusion criteria. A final limitation is that the determination of hemolysis was made by laboratory personnel and recorded as “present or absent” as opposed to “mild, major or severe hemolysis.” This may have introduced variation over time and across institutions as to what results qualified as hemolyzed; however, it would not necessarily have affected this decision rule’s sensitivity. Lastly, since patients with renal insufficiency, malignancy, etc were excluded, we do not know the incidence of pseudohyperkalemia in these patients for comparison.

## CONCLUSION

Our results suggest that in appropriately selected adult ED patients < 65 years of age with normal renal function, no hematologic malignancy, and who are not on potassium-modulating drugs, there is little to no risk of true hyperkalemia. Further studies should be done for confirmation of the criteria’s applicability in other settings and potential expansion to older patients with normal GFR.

## Figures and Tables

**Table 1:** Selection criteria to predict the absence of truly elevated potassium levels on repeat blood draw.

Selection criteria	
a.	18 ≤ age < 65
b.	eGFR ≥ 60
c.	HCO_3_ > 20
d.	CPK < 500 (if measured)
e.	Not taking potassium-modulating drugs at time of presentation (spironolactone, triamterene, aldactone, ACE-inhibitor)
f.	Without known hematologic malignancy
g.	Not administered albuterol, HCO_3_, insulin, furosemide or potassium binding medications after the first potassium draw and before the second potassium draw

*eGFR*, estimated glomerular filtration rate;*CPK*, creatine phosphokinase.
